# The type III secretion chaperone SctY may shield the hydrophobic export gate-binding C-terminus of its substrate SctX

**DOI:** 10.1107/S2059798323003248

**Published:** 2023-05-19

**Authors:** Dominic Gilzer, Julia L. Kowal, Franziska Flottmann, Hartmut H. Niemann

**Affiliations:** a Department of Chemistry, Bielefeld University, Universitaetsstrasse 25, 33615 Bielefeld, Germany; University of Sâo Paulo, Brazil

**Keywords:** helix bending, protein–protein interactions, type III secretion systems, YscX, YscY, AscX, *Aeromonas hydrophila*, *Yersinia enterocolitica*, type III secretion chaperone SctY

## Abstract

Gram-negative bacteria such as *Aeromonas* and *Yersinia* spp. have developed mechanisms to inhibit the immune defense of their host. Effector proteins are directly injected into the host cytoplasm from the bacterial cytosol via type III secretion systems (T3SSs), where they modulate the cytoskeleton and signaling of the cell. Assembly of, and secretion via, T3SSs is tightly regulated by a number of bacterial proteins, including SctX (AscX in *Aeromonas*), the secretion of which is essential for T3SS function. Here, crystal structures of AscX in complex with SctY chaperones from *Yersinia* or *Photorhabdus* spp. carrying homologous T3SSs are described. There are crystal pathologies in all cases, with one crystal form diffracting anisotropically and the other two exhibiting strong pseudotranslation. The new structures reveal that the positioning of the substrate is very similar on different chaperones. However, the two C-terminal SctX helices that cap the N-terminal tetratricopeptide repeat of SctY shift and tilt depending on the identity of the chaperone. Moreover, the C-terminus of the α3 helix of AscX exhibits an unprecedented kink in two of the structures. In previous structures, the C-terminus of SctX protrudes beyond the chaperone as a straight helix: a conformation that is required for binding to the nonameric export gate SctV but that is unfavorable for binary SctX–SctY complexes due to the hydrophobicity of helix α3 of SctX. A kink in helix α3 may allow the chaperone to shield the hydrophobic C-terminus of SctX in solution.

## Abstract 2

SctX proteins are secreted substrates and are essential components of type III secretion systems. The dedicated chaperone SctY escorts SctX proteins to the secretion machinery, where the hydrophobic helix at the C-terminus of SctX mediates recognition by the export apparatus. New crystal structures suggest that in the absence of the export gate this hydrophobic helix kinks and folds back onto the chaperone, presumably to shield it from the solvent.

## 1. Introduction

Pathogens and symbiotic bacteria need to evade the immune response of their host for survival. The type III secretion systems (T3SSs or injectisomes) that several Gram-negative species employ to inject effector proteins into the host-cell cytoplasm represent one way in which this can be achieved (for reviews, see Dewoody *et al.*, 2013 ▸; Portaliou *et al.*, 2016 ▸). Substrates of the T3SS are often escorted to the export apparatus by a chaperone, which maintains the partially unfolded conformation of the substrate (Letzelter *et al.*, 2006 ▸). After delivery to the injectisome, ATP hydrolysis at the cytosolic type III secretion ATPase complex separates the substrate from the chaperone, unfolding the former (Akeda & Galán, 2005 ▸). Afterwards, the substrate is funneled through the injectisome in a linearized or partially helical state (Miletic *et al.*, 2021 ▸).

Secretion through the injectisome is strictly regulated. After recruiting the export apparatus and ATPase complex to the membrane-embedded basal body, components of the inner rod and the needle are secreted (SctI and SctF in the unified nomenclature). After completion of the needle, a substrate-specificity switch enables the recognition and export of hydrophobic translocator proteins, which insert into the plasma membrane of the host. Finally, effectors are injected into the host cell via the newly formed pore (Portaliou *et al.*, 2016 ▸). While most substrates fall into one of these three categories, the SctX protein, which is only found in the Ysc family of T3SSs, is secreted after needle completion but before secretion of the translocators (Diepold *et al.*, 2012 ▸).

Within the cytosol, SctX is bound by its chaperone SctY (Iriarte & Cornelis, 1999 ▸; Day & Plano, 2000 ▸). Our recently published crystal structure of the *Yersinia* YscX–YscY complex revealed an entwined binding mode in which the substrate simultaneously binds the concave groove as well as the N-terminal face of the tetratricopeptide repeat (TPR)-containing chaperone (Gilzer *et al.*, 2022 ▸). While their function remains enigmatic, both SctX and SctY are essential for the formation of a secretion-competent T3SS (Iriarte & Cornelis, 1999 ▸; Day & Plano, 2000 ▸; Bröms *et al.*, 2005 ▸). SctX and SctY engage the export apparatus via the C-terminus of the substrate, which anchors the substrate between two protomers of the SctV nonamer (Gilzer *et al.*, 2022 ▸). The importance of this interaction *in vivo* and structural superpositions indicate a regulatory role of SctX in the export of early substrates (Diepold *et al.*, 2012 ▸; Gilzer *et al.*, 2022 ▸)

While the binding mode is similar between SctX and SctY from different species, as judged by their ability to form heterologous complexes in yeast two-hybrid assays, deletions of *yscX* or *yscY* in *Yersinia* could not be complemented using homologous *sctX* and *sctY* genes from other species (Bröms *et al.*, 2005 ▸; Gurung *et al.*, 2018 ▸). To investigate whether other SctX and SctY proteins exhibit distinct structural features, we crystallized heterologous complexes of the *Aeromonas* substrate AscX with different chaperones.

## 2. Materials and methods

### 2.1. Molecular cloning

The *ascX* gene from *Aeromonas hydrophila* AH3 lacking the leading 30 residues (*ascX*_31_) was cloned into a pETM-40 vector for expression as a fusion with maltose-binding protein (MBP). The source DNA was obtained from Invitrogen as a synthetic construct. To obtain the further truncated *ascX*_49_ (coding for residues 49–121) in the same vector, ‘round-the-horn’ mutagenesis was used. The *lscY* gene from *Photorhabdus luminescens* subsp. *laumondii* TT01 was similarly cloned from genomic DNA into the first multiple cloning site of pACYCDuet-1 for expression as an N-terminally hexa­histidine-tagged protein. *Yersinia enterocolitica* W22703 *yscY* was cloned as described previously (Gilzer *et al.*, 2022 ▸).

### 2.2. Expression and purification of SctX–SctY

Complexes of the substrates AscX_31_ or AscX_49_ with the chaperones YscY or LscY were prepared as described previously for the YscX_32_–YscY and YscX_50_–YscY complexes (Gilzer *et al.*, 2022 ▸). Briefly, AscX fused to an N-terminal MBP tag was coexpressed with the chaperone carrying an N-terminal hexahistidine tag in *Escherichia coli* BL21 (DE3) cells. After lysis of the cells and centrifugation, the supernatant was loaded onto amylose resin (10 ml Amylose Resin High Flow, New England Biolabs). The MBP tag was cleaved by adding Tobacco etch virus (TEV) protease. The SctX–SctY complex was then further purified from the supernatant via Ni–NTA affinity chromatography (5 ml Ni–NTA Agarose, Macherey-Nagel) to remove the MBP and finally applied onto a HiLoad 16/60 Superdex 75 pg gel-filtration column (Cytiva) using 20 m*M* Tris pH 8, 150 m*M* NaCl as the running buffer. Afterwards, the pure fractions were pooled, concentrated to approximately 10 mg ml^−1^ and frozen with 5 m*M* tris(2-carboxyethyl)phosphine (TCEP).

### 2.3. Analytical gel filtration

The proteins were thawed on ice, diluted to 2 mg ml^−1^ and incubated for 30 min. Afterwards, samples were loaded onto a Superdex 75 10/300 GL column (Cytiva) run at a flow rate of 0.5 ml min^−1^ with 20 m*M* Tris pH 8, 150 m*M* NaCl as the running buffer. These conditions are very similar to those used previously to analyze YscX–YscY complexes. The buffer, flow rate and column size were identical, but Superdex 200 matrix was used because interactions with the ∼500 kDa SctV nonamer were being studied. YscX–YscY was loaded at a concentration of 10 µ*M* (∼0.25 mg ml^−1^; Gilzer *et al.*, 2022 ▸).

### 2.4. Reductive methylation of AscX_49_–LscY

AscX_49_–LscY purified by gel filtration was modified following a previously described protocol (Gilzer *et al.*, 2022 ▸). The protein was rebuffered in HEPES buffer before adding 1.2 mg ml^−1^ borane–dimethylamine complex (97%, Alfa Aesar) and 1.2%(*v*/*v*) formaldehyde. Methylation proceeded overnight and was stopped by adding 250 m*M* Tris pH 8. The reaction was handled under a fume hood and measures were taken to protect eyes and skin. Finally, gel filtration was used to obtain pure methylated protein (AscX_49_–LscY^meth^).

### 2.5. Crystallization and data collection

Screens were prepared at 277 and 295 K with a Crystal Gryphon (Art Robbins Instruments) pipetting robot using commercially available screens. Crystals of AscX_31_–YscY were obtained at 10 mg ml^−1^ in 0.2 *M* sodium acetate, 0.1 *M* sodium citrate pH 5.5, 10%(*w*/*v*) PEG 4000 at 277 K and were reproduced under the same conditions but using protein at 7.5 mg ml^−1^. Drops consisted of 0.33 µl reservoir solution and 0.66 µl protein solution and were incubated over 60 µl reservoir solution. Crystals were harvested using 25%(*v*/*v*) glycerol as a cryoprotectant.

The initial crystals of AscX_49_–YscY grew in 0.1 *M* HEPES pH 7.5, 0.8 *M* NaH_2_PO_4_, 0.8 *M* KH_2_PO_4_ at 295 K using protein at 10 mg ml^−1^. Crystal growth was reproduced in similar conditions containing 0.6–0.9 *M* of either phosphate to give a total of 1.4–1.8 *M* dihydrogen phosphate. In optimization plates, the drop composition was 0.33 µl reservoir solution plus 0.66 µl protein solution. Crystals were harvested by transferring them to a cryoprotectant containing the reservoir solution supplemented with 20%(*v*/*v*) propylene glycol.

The initial AscX_49_–LscY^meth^ crystals were obtained from a screen using protein at 7.5 mg ml^−1^ and grew in 0.15 *M* KBr, 30%(*w*/*v*) PEG 2000 monomethyl ester (MME) at 295 K. Optimized crystals grew using 5 mg ml^−1^ protein in 0.15 *M* KBr, 24–26%(*w*/*v*) PEG 2000 MME. As above, the drop consisted of 0.33 µl reservoir solution and 0.66 µl protein solution. Crystals were harvested and flash-cooled using a cryoprotectant solution consisting of 0.15 *M* KBr, 35%(*w*/*v*) PEG 2000 MME, 10%(*v*/*v*) PEG 400.

Diffraction data were collected on EMBL beamlines P14 (AscX_31_–YscY) and P13 (AscX_49_–YscY; Cianci *et al.*, 2017 ▸) at the PETRA III storage ring, DESY, Hamburg, Germany and on beamline ID23-1 (AscX_49_–LscY^meth^) at ESRF, Grenoble, France (Mueller-Dieckmann *et al.*, 2015 ▸). Measurements were carried out using local installations of *MXCuBE*2 (Oscarsson *et al.*, 2019 ▸) or *MXCuBE*3.

Diffraction images for all structures are available via the SBGrid Data Bank (Meyer *et al.*, 2016 ▸) and additionally via the ESRF Data Portal for data collected at the ESRF. DOIs for the data sets are given in Table 1 ▸.

### 2.6. Data reduction, model building and refinement

Indexing, integration and scaling took place in *XDS* and *XSCALE* (Kabsch, 2010 ▸) via *XDSGUI*. Molecular replacement was performed using YscX_50_–YscY (PDB entry 7qih; Gilzer *et al.*, 2022 ▸) as a model in *Phaser* (McCoy *et al.*, 2007 ▸) with the tNCS option disabled. Iterative model-building cycles in *Coot* (Emsley *et al.*, 2010 ▸) and refinement using *phenix.refine* (Liebschner *et al.*, 2019 ▸; Afonine *et al.*, 2012 ▸) generated the final models. All structures were refined with TLS parameters (‘find TLS’ option) and both the stereochemistry and the ADP weights were optimized. When model building and refinement were almost complete, the resolution cutoff for all data sets was determined with paired refinement (Karplus & Diederichs, 2012 ▸) using complete cross-validation over all 20 *R*_free_ flags as implemented in *PAIREF* (Malý *et al.*, 2020 ▸, 2021 ▸). As the AscX_31_–YscY data were strongly anisotropic, an anisotropic resolution cutoff was applied to unmerged data from the CORRECT step of *XDS* with the *STARANISO* server (Tickle *et al.*, 2018 ▸). Scaling statistics for isotropically truncated data from *XSCALE* and anisotropically truncated data from the *STARANISO* server are given in Table 1 ▸. The use of anisotropically truncated data decreased *R*_free_ by about ∼1.5% upon refinement without rebuilding and finally allowed the placement of some additional terminal residues. The AscX_49_–YscY model was refined with noncrystallographic symmetry (NCS) restraints. The AscX_49_–LscY^meth^ data were collected at the peak wavelength of bromine and showed signs of radiation damage after as few as 800 images (160° rotation). To find a compromise between sufficient anomalous completeness and minimizing the negative effects of radiation damage, only 1000 of the 1800 collected images were included. The AscX_49_–LscY^meth^ model was refined against *I*(+) and *I*(−) using NCS restraints. Bromide ions were located in the anomalous difference map and were refined as anomalous scatterers. Figures and alignments were generated in *PyMOL*. R.m.s.d. values were calculated using C^α^ atoms without outlier rejection in *PyMOL*. Unless stated otherwise, the entire residue range was aligned. Coordinates and structure factors are available from the PDB (entries 8ara, 8arb and 8arc).

## 3. Results

### 3.1. Structures of AscX in complex with YscY

The type III secretion substrate SctX binds its chaperone at two distinct sites: (i) its α1 helix interacts with the concave groove of SctY and (ii) the two C-terminal helices α2 and α3 cover the large hydrophobic surface formed by the N-terminal TPR of the chaperone (Gilzer *et al.*, 2022 ▸). The C-terminus of SctX extends beyond the complex and is necessary for its recognition by the export-gate protein SctV. Yeast two-hybrid experiments established the ability of cross-species binding between SctX and SctY from a different species (hereafter Sct′Y) proteins, for instance allowing *Y. enterocolitica* YscY to act as chaperone for *A. hydrophila* AscX (Gurung *et al.*, 2018 ▸). We co-purified and crystallized two different complexes of AscX and YscY, with the substrate truncated N-terminally to residue 30 (AscX_31_) or residue 48 (AscX_49_). The latter construct was designed to start with the α1 helix. Data statistics are reported in Table 1 ▸.

AscX_49_–YscY crystallized with four complexes in the asymmetric unit. As the density of chain *E* was poor, helices α5 and α6 of this YscY monomer were not included in the final model. Refinement stalled at high *R*_work_ and *R*_free_ values of 29.12% and 32.62%, respectively. We attribute this to the presence of strong translational noncrystallographic symmetry (tNCS), indicated by a Patterson peak at (0.000, 0.315, 0.500) with a height of 49% of the origin peak. Such pseudotranslation causes a high fraction of weak reflections and has been reported to result in high *R* factors (Vajdos *et al.*, 1997 ▸; Barends & Dijkstra, 2003 ▸; Neumann *et al.*, 2022 ▸).

To reduce the risk of space-group misassignment, we solved the structure in space group *P*1 with 16 heterodimers per asymmetric unit and used *Zanuda* (Lebedev & Isupov, 2014 ▸) on this structure, which again suggested *C*222_1_ with the same packing as the most likely space group. The data showed neither clear signs of radiation damage nor strong anisotropy. To avoid overestimating the resolution, we determined the resolution cutoff with completely cross-validated paired refinement in *PAIREF*. Taken together, the strong pseudotranslation appears to be the most likely cause of the high *R* factors.

**Figure 1:**
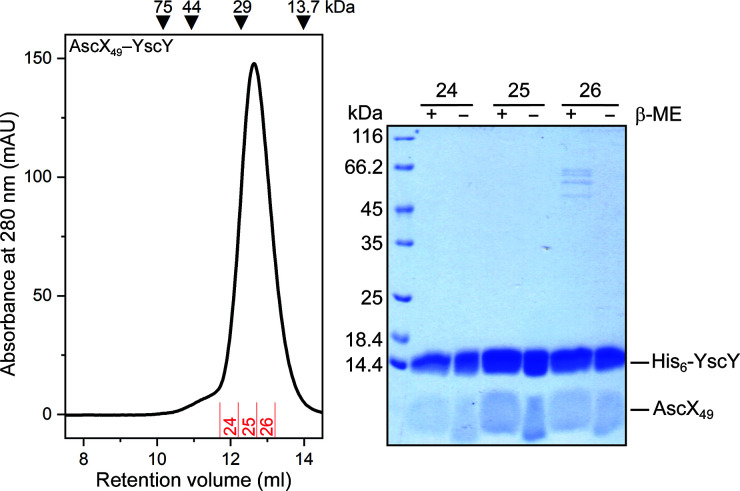
Analytical size-exclusion chromatography of AscX_49_–YscY. 200 µl of a 2 mg ml^−1^ protein solution was loaded onto a Superdex 75 10/300 GL column (Cytiva). The protein eluted as a single peak at the expected molecular weight. Fractions were collected and analyzed via reducing and nonreducing SDS–PAGE. The two bands were allocated to YscY and AscX_49_. β-ME, β-mercaptoethanol.

The four AscX_49_–YscY complexes in the asymmetric unit roughly follow pseudo-222 symmetry and the C-terminal α3 helices of each AscX bind each other in a four-helix bundle. Some pairs of the four AscX_49_–YscY complexes are related by approximate twofold rotational symmetry, while others substantially deviate from pure twofold rotations (Supplementary Table S1) as determined by *LSQKAB* (Kabsch, 1976 ▸). The *PISA* server (Krissinel & Henrick, 2007 ▸) predicts either a complex containing two tetramers of the heterodimer or one such tetramer as a stable assembly in solution. Neither was observed, as AscX_49_–YscY showed a dominant peak during gel filtration at the expected retention volume for a 1:1 complex (Fig. 1 ▸). All four complexes in the asymmetric unit show a similar structure and superimpose well (Supplementary Fig. S1*c* ).

**Figure 2:**
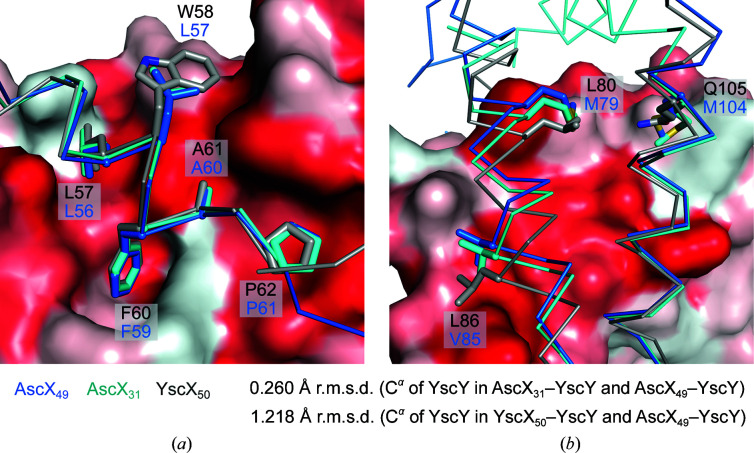
Binding interfaces of AscX and YscY. Superpositions of AscX_49_–YscY (blue, PDB entry 8arb), AscX_31_–YscY (cyan, PDB entry 8ara) and YscX_50_–YscY (gray, PDB entry 7qih) are shown with the substrate depicted as a ribbon and sticks. The surface of the AscX_49_–YscY chaperone is colored according to its hydrophobicity from red (highly hydrophobic residues) to white (hydrophilic residues) (Eisenberg *et al.*, 1984 ▸). (*a*) The α1 binding site is identical, except for the sequence difference Trp58 in YscX versus Leu57 in AscX. (*b*) Three sequence differences in the α2 and α3 helices are shown, which occur at the edge of the binding interface between substrate and chaperone.

Similar to our previously published structures of YscX bound to YscY, AscX is recognized by YscY at two distinct binding interfaces: (i) the α1 helix of AscX binds to the hydrophobic groove of the TPR chaperone and (ii) the C-terminal α2 and α3 helices of the substrate cap the N-terminal TPR of YscY, thereby masking a large hydrophobic surface. The amphipathic α1 helix is highly conserved between AscX and YscX, with hydrophobic side chains binding into well defined pockets on the surface of the chaperone (Fig. 2 ▸ *a*). Instead of Trp58 of YscX, AscX carries the smaller Leu57, which does not affect the binding mode. At the second binding interface, three relevant sequence differences (Leu80, Leu86 and Gln105 in YscX compared with Met79, Val85 and Met104 in AscX) separate the two substrates. The effect on chaperone binding is minimal, however, since all three residues are situated at the edge of the interface, as underscored by their poor sequence conservation (Fig. 2 ▸ *b*; Gilzer *et al.*, 2022 ▸).

**Figure 3:**
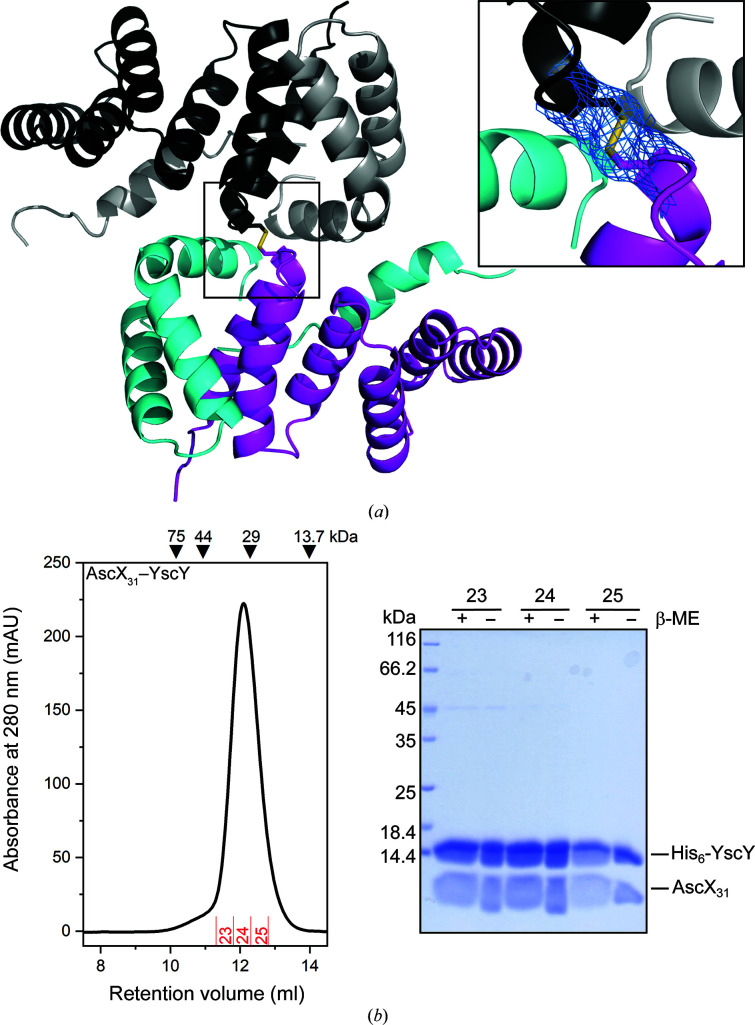
Structure of the AscX_31_–YscY complex. (*a*) Cartoon representation of the AscX_31_–YscY crystal structure. One heterodimer is colored cyan (AscX_31_) and magenta (YscY), while the other is shown in gray (AscX_31_) and black (YscY). The inset highlights the covalent linkage between Cys23 of the two YscY chains in the asymmetric unit. The 2*mF*_o_ − *DF*_c_ density is contoured at the 1σ level. (*b*) Analytical size-exclusion chromatography using a Superdex 75 10/300 GL column (Cytiva) produced a single peak for AscX_31_–YscY at the expected weight for a 1:1 complex. No cross-linked species were observed on a polyacrylamide gel.

Crystals of AscX_31_–YscY formed within 3–5 weeks and were difficult to reproduce, often only growing unsystematically in a few near-identical conditions. The AscX_31_–YscY complex crystallized with two almost identical complexes in the asymmetric unit (Supplementary Fig. S1*a* ). Despite AscX containing all amino acids after residue 30, the electron density only supported model building from Arg45, indicating that the N-terminus is disordered. This is similar to the structure of YscX_32_–YscY (PDB entry 7qii), in which YscX was only resolved from Leu47 (Gilzer *et al.*, 2022 ▸). Interestingly, the two YscY monomers are covalently linked via a disulfide bond between Cys23 of both chains (Fig. 3 ▸ *a*) despite the presence of 5 m*M* TCEP as a reducing agent in the protein solution used for crystallization. The presence of TCEP might have impeded the oxidation-dependent crystallization of AscX_31_–YscY, resulting in long growth times and low reproducibility. Superimposing the YscY monomers using *LSQKAB* (Kabsch, 1976 ▸) revealed a purely rotational relation between the two chains, with an angle of 90.1° between the twofold axis and the centroid vector and a rotation of χ = 179.0°. Furthermore, AscX_31_–YscY did not show oligomerization when subjected to gel filtration, where it eluted as a single peak at the retention volume expected for a 1:1 heterodimer (Fig. 3 ▸ *b*). Neither the structure of the homologous YscX_50_–YscY nor that of YscX_32_–YscY (PDB entries 7qih and 7qii, respectively) exhibited the formation of a disulfide bond, since the two Cys23 residues are separated by 6.3 Å in YscX_50_–YscY and by 8.6 Å in YscX_32_–YscY. The contact between the two chains is instead closest at Leu19.

**Figure 4:**
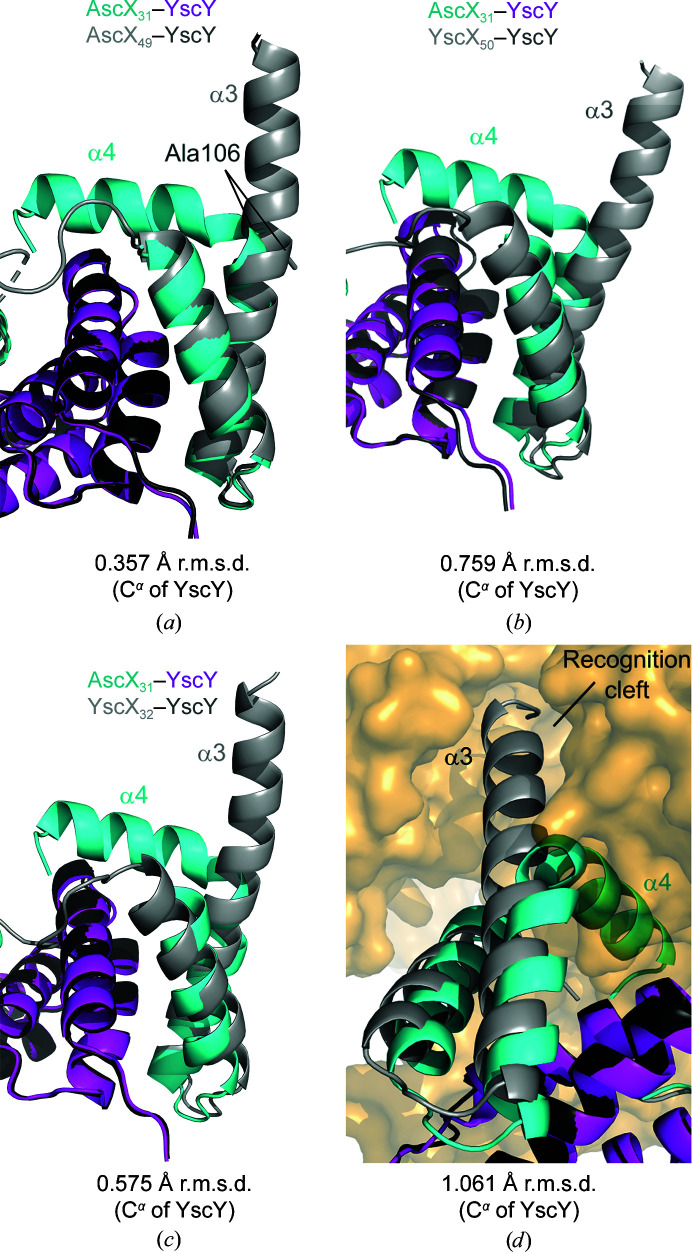
Bending of the α3 helix does not occur in previous crystal structures. Superpositions of AscX_31_–YscY with (*a*) AscX_49_–YscY (PDB entry 8arb), (*b*) YscX_50_–YscY (PDB entry 7qih), (*c*) YscX_32_–YscY (PDB entry 7qii) and (*d*) YscX_32_–YscY engaging the cytosolic domain of the export gate YscV (orange surface; PDB entry 7qij) are shown.

Interestingly, both AscX monomers showed a bend in the α3 helix at Ala106 despite the high propensity of alanine for the formation of helices. This helix bending was not observed for the lower resolution crystal structure of the shorter AscX_49_–YscY construct, despite an otherwise identical structure to AscX_49_–YscY (Fig. 4 ▸ *a*). The α3 kink has not been observed in any YscX–YscY binary complex structure (Figs. 4 ▸ *b* and 4 ▸ *c*) and an extended conformation of the SctX C-terminus is necessary for recognition of the substrate by the export gate SctV (Fig. 4 ▸ *d*).

### 3.2. LscY causes a shift of the α2 helix in AscX

To evaluate whether the identity of the chaperone influences the binding to the substrate, we also attempted to co-crystallize AscX with a different chaperone to YscY. We were unable to express *A. hydrophila* His_6_-AscY in *E. coli*, but were able to solubly co-express AscX with *P. luminescens* subsp. *laumondii* His_6_-LscY. The AscX_49_–LscY complex was reductively methylated to enable crystallization and diffracted to approximately 2 Å resolution (Table 1 ▸). The asymmetric unit contains two heterodimers related via a tNCS vector (0.000, 0.500, 0.500) with a height of 83% compared with the origin Patterson peak. As for the AscX_49_–YscY structure, we assume that the high final *R*_free_ value of 28.62% is due to the large proportion of weak reflections caused by pseudotranslation (Vajdos *et al.*, 1997 ▸; Barends & Dijkstra, 2003 ▸; Neumann *et al.*, 2022 ▸).

**Figure 5:**
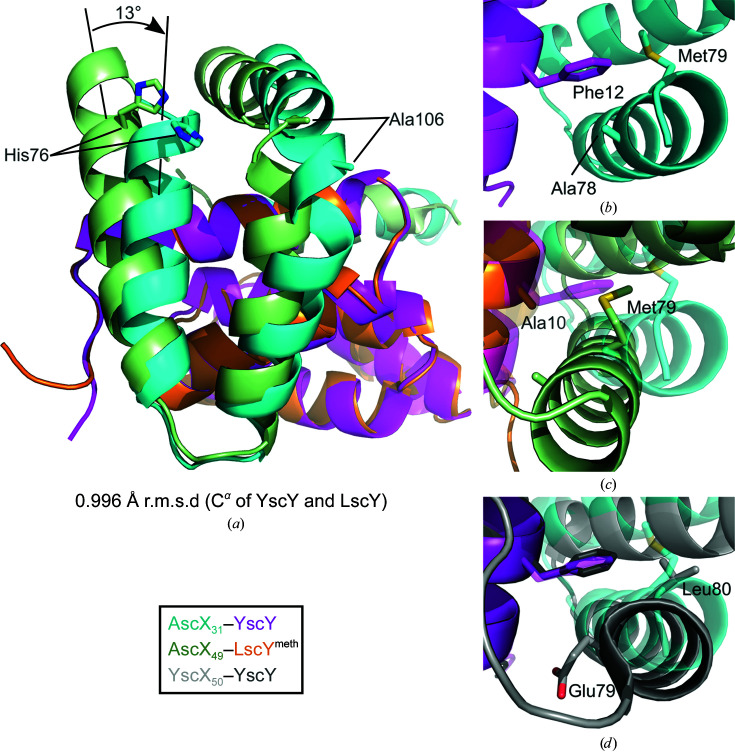
The C-terminal helices of AscX shift depending on the chaperone. (*a*) Superposition of AscX_31_–YscY and AscX_49_–LscY^meth^ calculated using the chaperones. AscX_31_ is depicted in cyan, YscY in magenta, AscX_49_ in green and LscY in orange. The rotation of 13° between the α2 helices was determined from the distance between the His76 residues (distance of 6.1 Å at the C^α^ atom) at the N-terminal end and the Glu88 residues (distance of 1.1 Å) at the C-terminal end of the helix. (*b*) Top view onto the C-terminal helices of AscX_31_ in complex with YscY. (*c*, *d*) Top view of the α2 helix in AscX_49_–LscY^meth^ and YscX_50_–YscY (PDB entry 7qih) aligned with AscX_31_–YscY.

The two AscX_49_–LscY^meth^ dimers in the asymmetric unit are virtually identical (Supplementary Fig. S1*b* ) and both exhibit the α3 bend in the substrate as described for AscX_31_–YscY. Notably, the bend occurs at the same residue, Ala106 (Fig. 3 ▸ *a*). Compared with AscX_31_–YscY, the α2 helix of AscX_49_ in complex with LscY is shifted towards the N-terminal end of the first α-helix of the chaperone and is positioned closer to the TPR (Figs. 5 ▸ *b* and 5 ▸ *c*). While the C-terminal end of the α2 helix superimposes well between the two complexes, the N-terminal end is rotated by about 13°, resulting in a shift of about 6.1 Å of His76 at the N-terminal end of the helix. Furthermore, the α3 helix appears to be rotated by about 10° in a similar fashion, with Ala106 being displaced by approximately 5.6 Å. A possible explanation for this shift is the presence of Phe12 in YscY instead of Ala10 in LscY, which causes steric conflicts with Met79 in the α2 helix of AscX. Consequently, the small side chain of Ala78 of AscX can pack more tightly against the N-terminal face of LscY than against YscY (Fig. 5 ▸ *c*). No other major differences were observed in the N-terminal surfaces of YscY and LscY.

In AscX_31_–YscY, the distance between the phenylalanine side chain and the terminal methyl group of Met79 amounts to 3.9 Å. The distance between Ala10 of LscY and Met79 of AscX in the AscX_49_–LscY complex was measured at a similar 4.1 Å. Moreover, the complex of YscX_50_ and YscY (PDB entry 7qih) exhibits a very similar position of the α2 helix of the substrate when compared with AscX_31_–YscY (Fig. 5 ▸ *d*), indicating that the smaller Leu80 in YscX is also pushed away from the face of the chaperone by the bulky phenylalanine side chain. Ala10 is conserved between LscY and AscY, hinting that the α2 shift would also occur when AscX is complexed by its native substrate. This is consistent with the higher sequence conservation between AscY and LscY (60.19% over the entire sequence or 74.36% for the first TPR, residues 1–39) than between AscY and YscY (53.15% for the full-length protein or 58.97% for the first TPR).

To investigate whether the α2 shift observed for AscX–LscY may also occur in the AscX–AscY complex, we generated *AlphaFold*2 (Jumper *et al.*, 2021 ▸) models of all three AscX complexes via the *ColabFold* server (Mirdita *et al.*, 2022 ▸). In the predictions, the α3 helix is identical independent of which chaperone sequence was given (Supplementary Fig. S2). In contrast, the α2 helix of AscX in AscX–LscY and AscX–AscY is shifted outwards compared with the prediction for AscX–YscY (Supplementary Fig. S2*d* ), similar to what was observed when comparing the crystal structures of AscX–YscY and AscX–LscY. The α2 shift and position are comparable between the crystal structures and the computational models (Supplementary Fig. S3).

## 4. Discussion

SctX is a substrate of virulent T3SSs but has an as yet undetermined function. The structure of YscX in complex with its chaperone as well as in the context of a ternary complex with the export gate has recently been established (Gilzer *et al.*, 2022 ▸). We now report three structures of *Aeromonas* AscX with heterologous chaperones that demonstrate the propensity of the protein to bend its C-terminal α3 helix. What causes the kink remains unclear. On one hand, it may be the result of crystal packing. On the other hand, the large fraction of hydrophobic residues in the C-terminus of SctX, combined with its positioning as an isolated solvent-exposed helix without an interaction partner, suggests that the C-terminal SctX helix may be bent in solution.

Superimposing a YscX_32_–YscY (PDB entry 7qii) or AscX_49_–YscY (PDB entry 8arb) heterodimer with extended SctX C-termini onto one heterodimer of the disulfide-bridged AscX_31_–YscY heterotetramer reveals that the C-terminus of SctX would collide with the α1 helix of AscX_31_ and with the third TPR of YscY in the neighboring heterodimer (Supplementary Fig. S4). An extended C-terminus of AscX would not fit into this configuration of the proteins, hence the α3 kink facilitates the assembly of the disulfide-bridged heterotetramer in the asymmetric unit of the AscX_31_–YscY crystals.

Similarly, overlays of AscX_49_–LscY with YscX_32_–YscY show collisions caused by extended α3 helices. The C-terminus of one superimposed YscX–YscY complex would occupy a void in the crystal and only cause minor clashes with the last histidine of the N-terminal hexahistidine tag of LscY and with the loop connecting helices α5 and α6 of the chaperone (Supplementary Fig. S5*a* ). These steric conflicts could most likely be alleviated by minor movements of the C-terminus of YscX or of the affected LscY residues. However, major clashes would arise between the extended C-termini of two symmetry-related substrate molecules (Supplementary Fig. S5*b* ). These superpositions imply that bending of the α3 helix of AscX is a prerequisite for packing of this crystal form.

**Figure 6:**
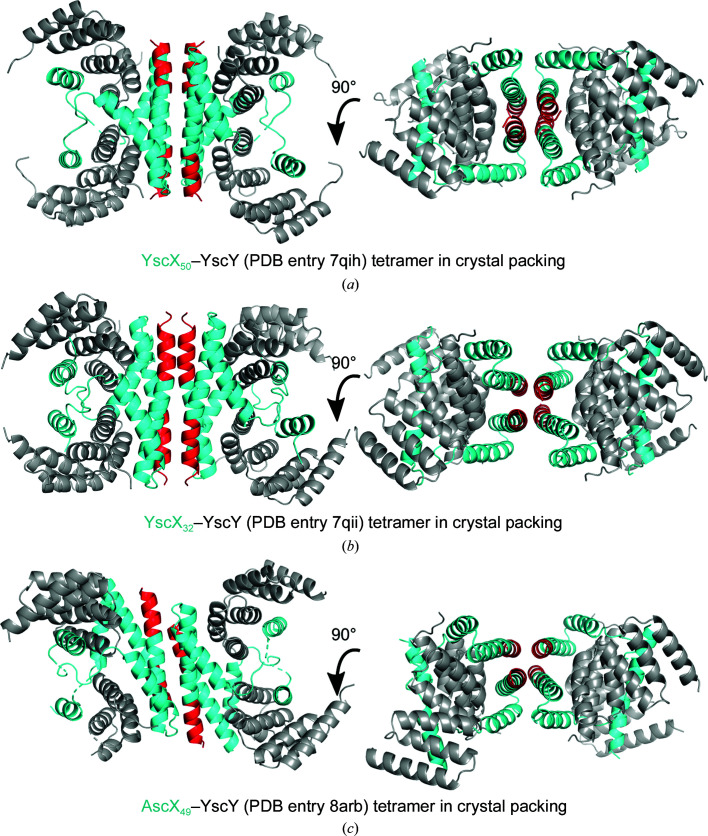
Crystallographic SctX–Sct′Y tetramers. Tetramerization via the C-terminal helices of SctX was observed in (*a*) YscX_50_–YscY (PDB entry 7qih), (*b*) YscX_32_–YscY (PDB entry 7qii) and AscX_49_–YscY (PDB entry 8arb) crystals. The chaperone is colored gray and the substrate is colored cyan, except for the C-terminal 12 residues, which are highlighted in red.

The C-terminal 12 residues of SctX proteins contain seven to eight hydrophobic residues, four of which are fully conserved (Leu115/116, Leu117/118, Leu118/119 and Val121/122 in AscX/YscX). When bound to the nonameric export-gate protein SctV, these hydrophobic residues of SctX occupy a hydrophobic groove formed mainly by two subdomains 4 (SD4) of adjacent SctV protomers. In the heterodimeric SctX–SctY complex without a binding partner, the extended C-terminus predominantly exposes hydrophobic residues to the solvent, which would be energetically unfavorable. In all three SctX–Sct′Y crystal forms with an extended C-terminus (YscX_50_–YscY, PDB entry 7qih; YscX_32_–YscY, PDB entry 7qii; AscX_49_–YscY, PDB entry 8arb), the C-terminal helices pack against each other to form antiparallel four-helix bundles (Fig. 6 ▸). While the exact geometry of these four-helix bundles differs between the three crystal structures, the hydrophobic residues of opposed SctX monomers are consistently involved in structures that are reminiscent of leucine zippers.

The recurring formation of (SctX–Sct′Y)_4_ assemblies via four-helix bundles might explain why YscX–YscY complexes usually elute from gel filtration in a complex profile with three distinct peaks corresponding to a heterodimer and presumably noncovalent dimers as well as tetramers of the YscX–YscY heterodimer (Gilzer *et al.*, 2022 ▸). Comparing the current and previous results appears valid as all gel-filtration experiments used very similar experimental conditions (see Section 2). The main difference is an approximately tenfold higher protein concentration of AscX–YscY compared with YscX–YscY, which should favor oligomerization of the AscX-containing complexes. The α3 kink observed in the two AscX structures allows approximately the last 15 residues of the substrate to fold back onto a hydrophobic patch on helix α1 of SctY. A higher propensity for kink formation in helix α3 of AscX than in helix α3 of YscX could explain why AscX–Sct′Y complexes almost exclusively elute as heterodimeric complexes in gel filtration (Figs. 1 ▸ and 3 ▸).

## 5. Conclusion

This study describes three structures of the *Aeromonas* T3SS substrate AscX in complex with heterologous chaperones from the *Yersinia* (YscY) and *Photorhabdus* (LscY) injectisomes. The overall fold is conserved between the SctX–Sct′Y complexes. An exchange of Phe12 in YscY to Ala10 in LscY and AscY allows the repositioning of SctX helices α2 and α3. The resulting change in the overall shape of the SctX–Sct′Y complex may contribute to the inability of the *Aeromonas* and *Photorhabdus sctX* and *sctY* genes to complement *yscX* and *yscY* deletions in *Yersinia*. Finally, AscX displayed a propensity to form a kink in its C-terminal α3 helix. Consequently, the hydrophobic SctV-binding C-terminus of SctX is shielded by a hydrophobic patch on SctY instead of protruding from the chaperone. In the absence of nonameric SctV, the bent structure may represent the predominant conformation of SctY-bound SctX in solution.

## Supplementary Material

PDB reference: AscX_31_–YscY, 8ara

PDB reference: AscX_49_–YscY, 8arb

PDB reference: AscX_49_–LscY^meth^, 8arc

Supplementary Table and Figures. DOI: 10.1107/S2059798323003248/gi5041sup1.pdf

Raw diffraction data for PDB entry 8ara.: https://doi.org/10.15785/SBGRID/1007

Raw diffraction data for PDB entry 8arb.: https://doi.org/10.15785/SBGRID/1009

Raw diffraction data for PDB entry 8arc.: https://doi.org/10.15785/SBGRID/1008

Raw diffraction data for PDB entry 8arc.: https://doi.org/10.15151/ESRF-DC-1101658626

## Figures and Tables

**Table 1:** Data-collection and refinement statistics Values in parentheses are for the outer resolution shell. Data for AscX49–LscY^meth^ were processed with Friedel mates separated due to anomalous scattering.

	AscX_31_–YscY		AscX_49_–YscY	AscX_49_–LscY^meth^
PDB code	8ara		8arb	8arc
Data collection				
DOI for diffraction images	10.15785/SBGRID/1007		10.15785/SBGRID/1009	10.15785/SBGRID/1008, 10.15151/ESRF-DC-1101658626
Beamline	P14, DESY		P13, DESY	ID23-1, ESRF
Temperature (K)	100		100	100
Wavelength (Å)	0.9919		0.9919	0.9187
Space group	*P*2_1_2_1_2_1_		*C*222_1_	*C*2
Pathology	Anisotropic		Pseudotranslation Patterson peak with 50% at 0.000, 0.315, 0.500	Pseudocentering Patterson peak with 83% at 0.000, 0.500, 0.500
*a*, *b*, *c* (Å)	44.32, 94.85, 101.37		90.33, 160.56, 156.74	90.59, 34.22, 99.60
α, β, γ (°)	90, 90, 90		90, 90, 90	90, 101.81, 90
	*STARANISO*, anisotropic	*XSCALE*, isotropic		
Resolution (Å)	94.85–2.304 (2.417–2.304)	94.85–2.30 (2.36–2.30)	80.28–2.63 (2.70–2.63)	48.75–2.10 (2.15–2.10)
*R*_meas_	0.174 (0.938)	0.192 (1.949)	0.229 (4.831)	0.063 (1.295)
〈*I*/σ(*I*)〉	11.00 (2.99)	9.36 (1.30)	9.35 (0.61)	8.64 (1.23)
CC_1/2_	0.997 (0.859)	0.997 (0.526)	0.996 (0.239)	0.999 (0.588)
Completeness (spherical)	0.758 (0.290)	0.997 (0.769)	0.993 (0.926)	0.948 (0.947)
Completeness (elliptical)	0.931 (0.949)	n.a.	n.a.	n.a.
Multiplicity	9.35 (8.95)	9.15 (7.60)	24.86 (12.93)	1.99 (2.00)
Wilson *B* factor (Å^2^)	29.2		80.1	42.9
Refinement				
No. of reflections	14839		33394	32276
No. of free reflections	745		1664	1582
*R*_work_	0.1903		0.2912	0.2286
*R*_free_	0.2426		0.3262	0.2862
No. of atoms in asymmetric unit				
Total	2996		5313	2829
Protein	2877		5274	2805
Water	102		4	18
Other	17		35	6
Average *B* factors (Å^2^)				
Total	43		1049	68
Protein	44		109	68
Water	36		93	60
Other	40		111	69
R.m.s.d.				
Bond lengths (Å)	0.003		0.002	0.001
Bond angles (°)	0.470		0.258	0.437
Ramachandran statistics				
Favored (%)	98.25		96.35	100.00
Allowed (%)	1.75		3.65	0.00
Outliers (%)	0.00		0.00	0.00
Rotamer outliers (%)	0.00		0.18	0.00
