# Supplementary material for: The type III secretion chaperone SctY may shield the hydrophobic export gate-binding C-terminus of its substrate SctX
Source: Acta Crystallogr D Struct Biol. 2023 May 19;79(Pt 6):508–17. doi: 10.1107/S2059798323003248 (PMC10233624; doi:10.1107/S2059798323003248)
Supplement: Supplementary file 1 [file d-79-00508-sup1.pdf]

Acta Cryst D

STRUCTURAL BIOLOGY

Volume 79 (2023)

Supporting information for article:

The type III secretion chaperone SctY may shield the hydrophobic export gate-binding C-terminus of its substrate SctX

Dominic Gilzer, Julia L. Kowal, Franziska Flottmann and Hartmut H. Niemann

Table S1 Symmetry between complexes in the AU of the AscX$_{49}$:YscY crystal.

The angle χ describes the rotation needed to superimpose one chain on the other, with 180 ° describing a 2-fold rotation. An angle of 90 ° between this rotation axis and the centroid axis represents a pure rotation, while differing values indicate that a translation is needed for the superposition. All values were calculated using the Cα atoms over the entire residue range of the chaperone.

		**Chains C + D**	**Chains E + F**	**Chains G + H**
**Chains A + B**	χ / °	174.96	145.13	173.21
	Angle to centroid / °	89.71	72.71	89.73
**Chains C + D**	χ / °	-	175.88	136.44
	Angle to centroid / °	-	91.42	78.14
**Chains E + F**	χ / °	-	-	178.31
	Angle to centroid / °	-	-	89.13

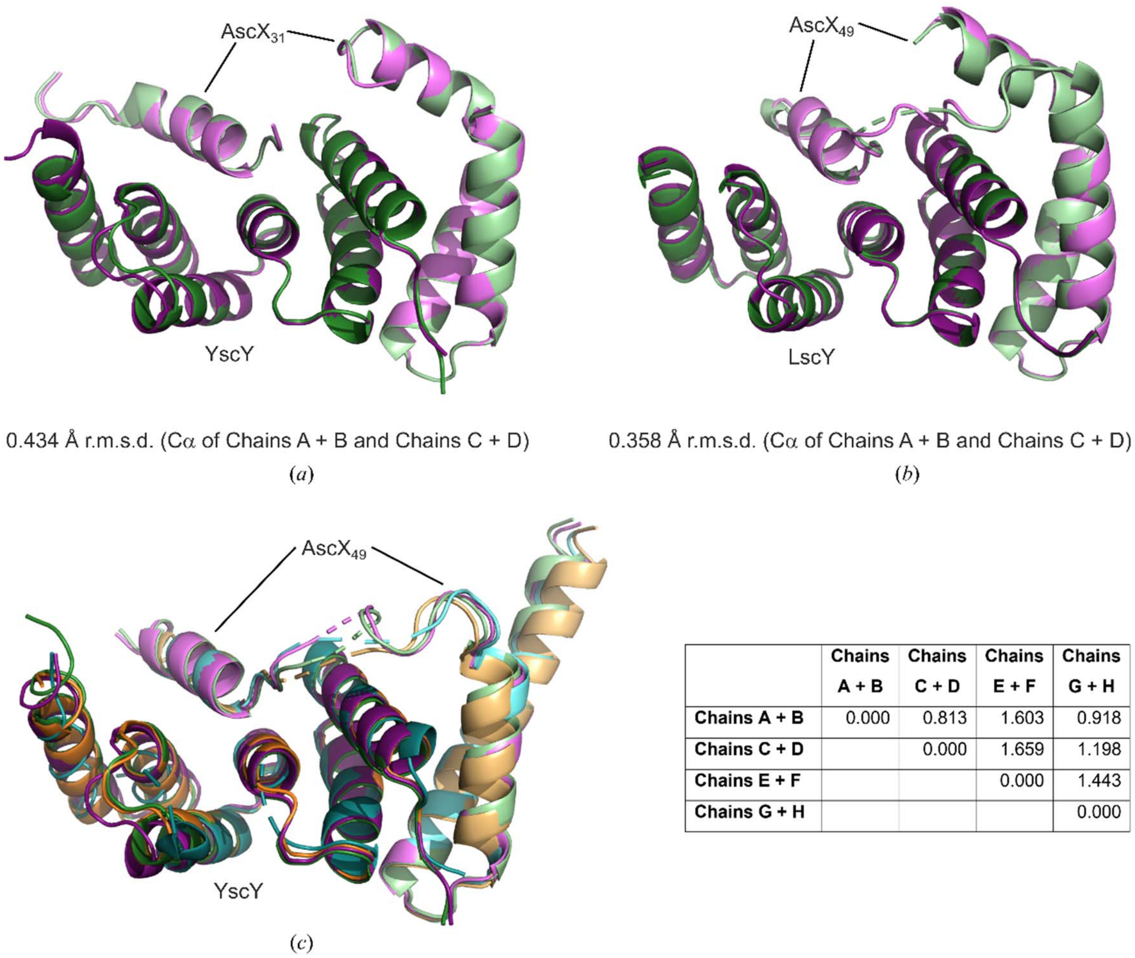

	Chains A + B	Chains C + D	Chains E + F	Chains G + H
**Chains A + B**	0.000	0.813	1.603	0.918
**Chains C + D**		0.000	1.659	1.198
**Chains E + F**			0.000	1.443
**Chains G + H**				0.000

Figure S1 Superposition of SctX:Sct'Y complexes within each crystal. (*a*) Two AscX$_{31}$:YscY (PDB entry 8ara), (*b*) two AscX$_{49}$:LscY^{meth} (PDB entry 8arc), and (*c*) four AscX$_{49}$:YscY (PDB entry 8arb) molecules are aligned in *PyMOL*. The r.m.s.d. value was calculated for Cα atoms without outlier rejection. For AscX$_{49}$:YscY, chain E exhibited notably worse electron density, resulting in an incomplete model lacking the C-terminus of YscY and worse r.m.s.d. values when superimposed onto other complexes in the AU.

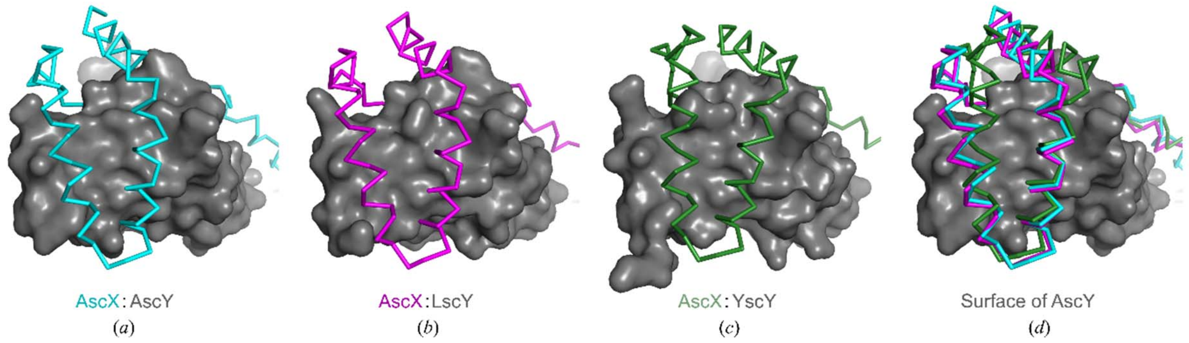

Figure S2 *AlphaFold2* predictions of AscX-containing complexes. Models were calculated using the *ColabFold* server supplying both sequences as separate polypeptide chains. Structures of (*a*) AscX:AscY, (*b*) AscX:LscY, and (*c*) AscX:YscY are shown with the chaperone as surface representation and AscX as ribbon. (*d*) Superposition of all three models on the surface of AscY.

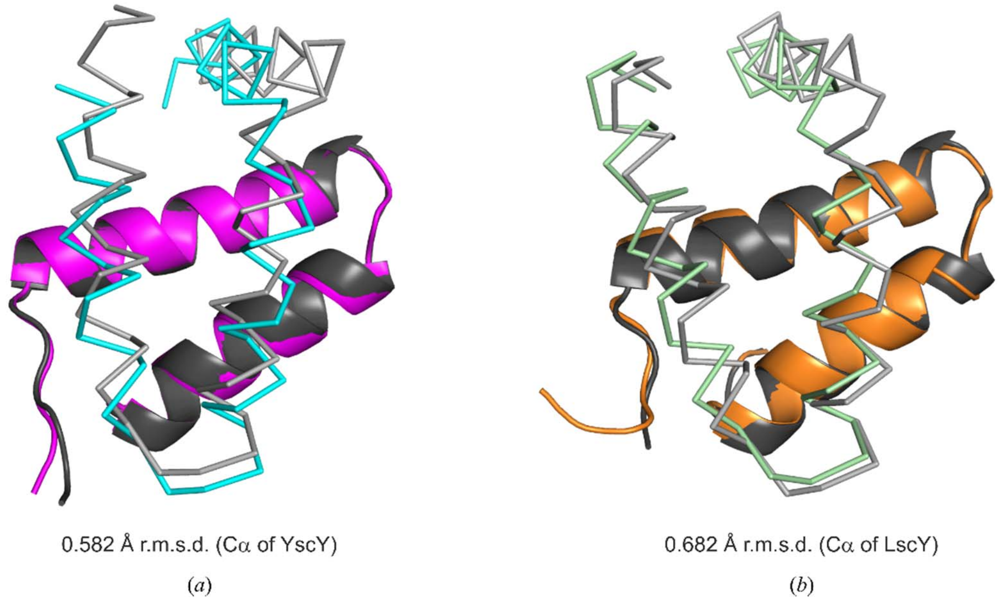

Figure S3 Comparison of AscX:SctY complexes with *AlphaFold2* predictions. The obtained crystal structures of (*a*) $AscX_{31}$:YscY (cyan and magenta), and (*b*) $AscX_{49}$:LscY (green and orange) were superimposed with their corresponding *AlphaFold2* predictions (AscX in light gray and chaperone in dark gray) in *PyMOL* using the chaperone molecule. For clarity, only the N-terminal TPR of the chaperone and the C-terminal helices of the substrate are shown.

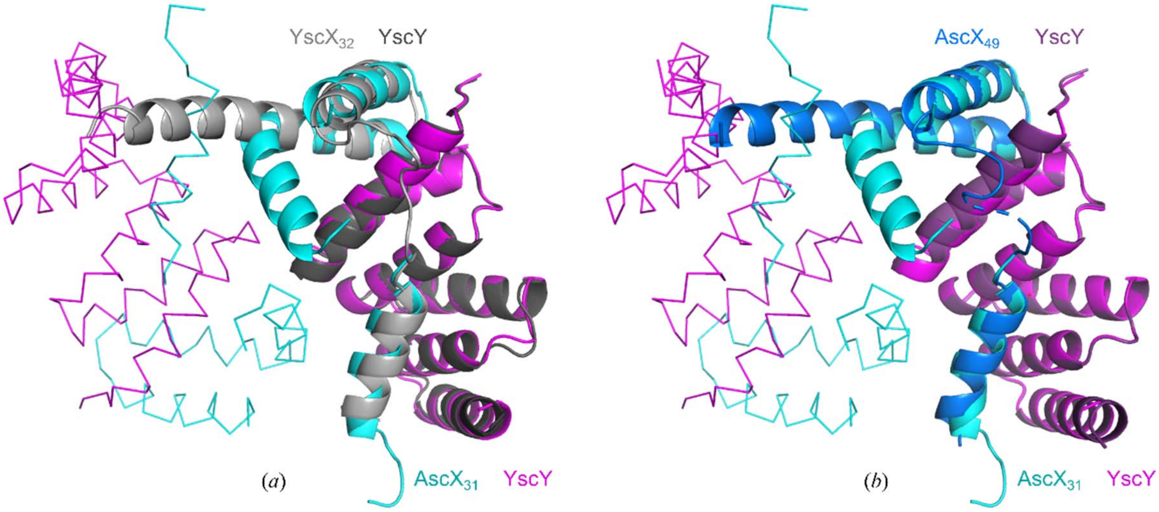

Figure S4 Clashing of extended SctX C-termini in the $AscX_{31}$:YscY crystal structure. The crystal structure of $AscX_{31}$:YscY (PDB entry 8ara; $AscX_{31}$ in cyan, YscY in magenta) was superimposed with a heterodimer of (*a*) $YscX_{32}$:YscY (PDB entry 7qii; $YscX_{32}$ in light gray, YscY in dark grey), or (*b*) $AscX_{49}$:YscY (PDB entry 8arb; $AscX_{49}$ in dark blue, YscY in violet). The second heterodimer of $AscX_{31}$:YscY is shown as ribbon model for clarity. An extended α3-helix as observed for $YscX_{32}$ and $AscX_{49}$ would clash with α1 of AscX and TPR3 of YscY in the $AscX_{31}$:YscY structure.

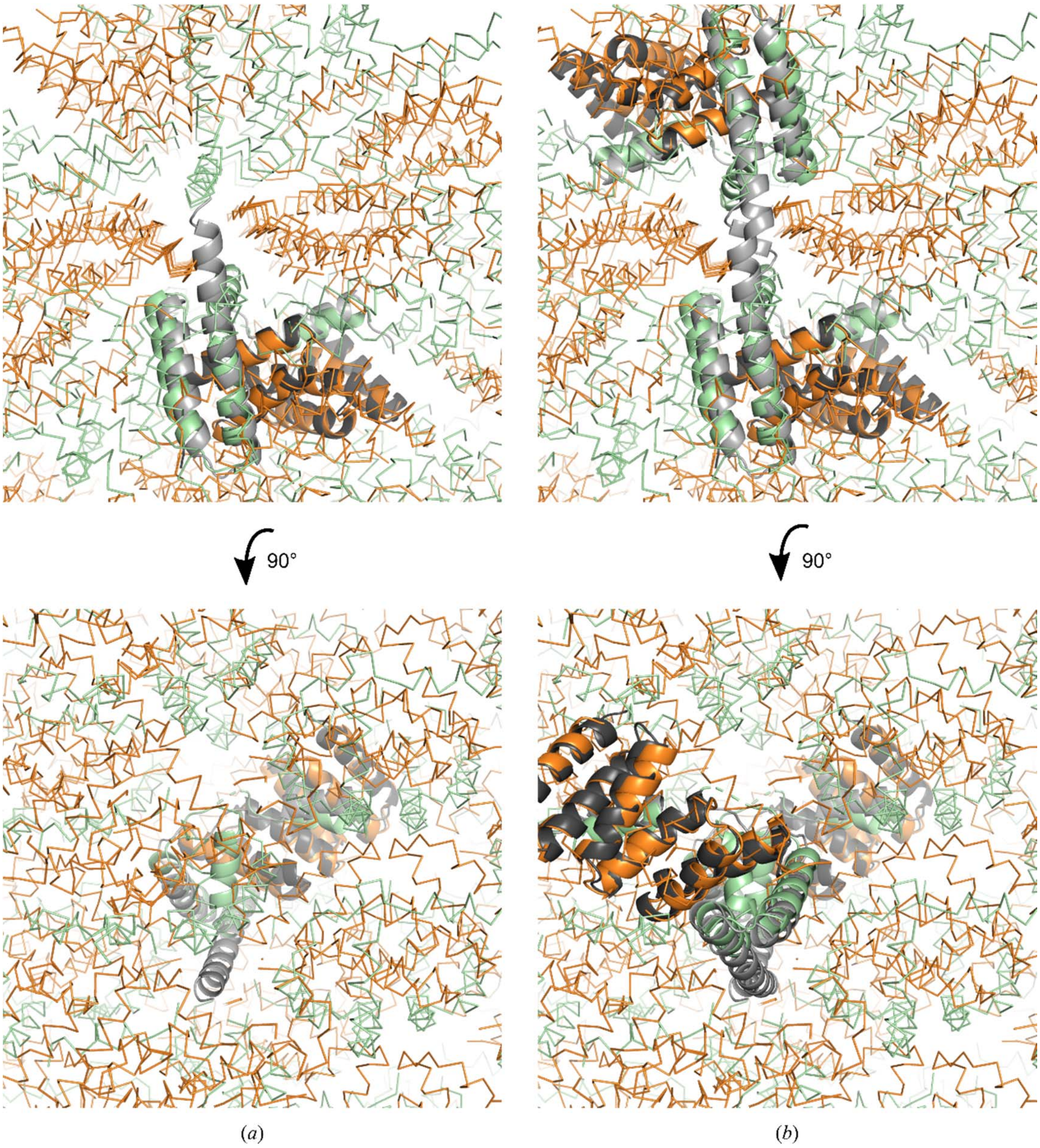

Figure S5 Placement of extended SctX C-termini in the $AscX_{49}$:LscY crystal structure. (*a*) The crystal structure of $AscX_{49}$:LscY (PDB entry 8arc; $AscX_{49}$ in green, LscY in orange) was superimposed with a heterodimer of $YscX_{32}$:YscY (PDB entry 7qii; $YscX_{32}$ in light gray, YscY in dark grey). The heterodimer of $AscX_{49}$:LscY used in the alignment is shown as cartoon, the remaining ones are shown as ribbons. A potential extended AscX C-terminus would hardly clash with surrounding molecules in the crystal. The two views are rotated by 90° about one axis. (*b*) A second $YscX_{32}$:YscY molecule was structurally aligned with a symmetry-related $AscX_{49}$:LscY heterodimer, resulting in clashes between the two extended C-termini of YscX.
